# A Palladium Catalyst Supported on Boron-Doped Porous Carbon for Efficient Dehydrogenation of Formic Acid

**DOI:** 10.3390/nano14060549

**Published:** 2024-03-20

**Authors:** Hui Liu, Mengyuan Huang, Wenling Tao, Liangliang Han, Jinqiang Zhang, Qingshan Zhao

**Affiliations:** 1 College of Chemistry and Chemical Engineering, Yantai University, Yantai 264005, China; lh@ytu.edu.cn (H.L.); 19558913013@163.com (M.H.); wenling0306@163.com (W.T.); hanll@ytu.edu.cn (L.H.); 2 School of Chemical Engineering, The University of Adelaide, North Terrace, Adelaide, SA 5005, Australia; jinqiang.zhang@adelaide.edu.au; 3 State Key Laboratory of Heavy Oil Processing, College of Chemistry and Chemical Engineering, China University of Petroleum (East China), Qingdao 266580, China

**Keywords:** formic acid, dehydrogenation, Pd catalyst, boron doping, porous carbon

## Abstract

Formic acid has emerged as a highly promising hydrogen storage material, and the development of efficient catalysts to facilitate its dehydrogenation remains imperative. In this study, a novel catalyst consisting of palladium nanoparticles supported on boron-doped porous carbon (Pd/BPC) was successfully synthesized to enable efficient hydrogen production through the dehydrogenation of formic acid. The impacts of the boron doping ratio, doping temperature, and palladium reduction temperature on the catalyst’s performance were systemically investigated. The results demonstrated the Pd/BPC catalyst synthesized with a carbon-to-boron ratio of 1:5 by calcination at 900 °C and subsequent reduction at 60 °C exhibited superior formic acid dehydrogenation performance, being 2.9 and 3.8 times greater than that of the Pd/PC catalysts without boron doping and commercial Pd/C, respectively. Additionally, the catalyst showed excellent cycle stability with no significant activity reduction after five consecutive cycles. Experimental and theoretical results reveal that boron doping not only facilitates the homogenous distribution of Pd nanoparticles but also induces a stronger support–metal interaction, thereby reinforcing the catalytic performance. This research is expected to provide valuable insights into the economically viable and efficient production of environmentally friendly hydrogen energy.

## 1. Introduction

With the escalating global energy demand and depletion of non-renewable sources, there is burgeoning interest in exploring alternative clean energy options [1,2]. Hydrogen has emerged as a promising contender owing to its potential as a sustainable and environmentally friendly fuel. However, one significant challenge impeding its widespread industrial application pertains to its storage and transportation risks [3,4]. To address this issue, precise control over hydrogen storage and release has become imperative for harnessing the full potential of hydrogen energy, particularly in applications such as hydrogen fuel cells. Among various media for storing hydrogen such as methanol, hydrazine hydrate, and ammonia borane, formic acid stands out as an appealing choice due to its high energy density, non-toxic nature, and stability at room temperature [5,6].

In order to efficiently extract hydrogen from formic acid, catalytic dehydrogenation of formic acid has been extensively studied and researchers worldwide are actively striving to develop novel catalysts. The majority of catalysts employed for the dehydrogenation of formic acid are predominantly based on noble metals, such as Pd, Ru, Ir, Pt, etc. [7,8,9,10]. For example, Huang’s group reported a ruthenium pincer complex immobilized on a fibrous silica nanosphere with good stability toward water, high pressures, and high temperatures in formic acid dehydrogenation [9]. An immobilized PN^3^P-Ir pincer catalyst supported onto a 3D fibrous silica nanosphere that contains a tetracoordinate aluminum site was further synthesized, exhibiting high TOF and TON [11]. Svetlana Ivanova’s group investigated the formic acid decomposition behavior of mono- and bimetallic Pd/Ru catalysts supported on graphitic C_3_N_4_ in both liquid- and vapor-phase conditions [12]. Nevertheless, these catalysts frequently encounter deactivation due to the generation of CO by-products during the reaction.

Carbon materials, such as activated carbon and carbon black, have long been considered good carriers of noble metal-based catalysts because of their high specific surface area, abundant pore structure, stable physical and chemical properties, and good electrical conductivity. Heteroatoms such as boron (B) and nitrogen (N) can be used as dopants to regulate the internal structure of carbon materials and improve their catalytic performance [13]. Koh et al. used an amino-functionalized SBA-15 molecular sieve as a carrier to enhance metal–carrier interaction and improve the catalytic activity of formic acid dehydrogenation [14]. Li et al. modified MSC-30 carrier by nitrogen doping using a low-temperature and high-temperature cascade treatment method and prepared a uniformly distributed palladium nanoparticle catalyst via the wet chemical reduction method, which showed good catalytic performance at 60 °C [15]. Kim et al. observed that nitrogen dopants can reduce the size of Pd particles and change the electronic state of Pd [16]. Lin et al. produced a series of N-doped carbon-dot-supported Pd nanoparticles, and these catalysts demonstrated exceptional catalytic performance in FA dehydrogenation without producing CO [17]. The incorporation of boron as a dopant has received limited attention to date.

Herein, a palladium catalyst supported on boron-doped porous carbon derived from petroleum asphalt (Pd/BPC) was successfully prepared, which efficiently modulates the property of the carbon substrate through the introduction of boron while preserving its porous structure and high specific surface area, effectively reinforcing the interaction between the support and Pd nanoparticles. The Pd/BPC catalyst exhibited promising outcomes in hydrogen production with enhanced activity and stability. In comparison to traditional palladium–carbon catalysts, the Pd/BPC catalyst offers a more cost-effective alternative for industries seeking sustainable solutions in hydrogen production or related fields.

## 2. Materials and Methods

### 2.1. Chemicals and Reagents

Most chemicals, including toluene, α-Fe_2_O_3_, hydrochloric acid, boron acid, palladium acetate, urea, sodium formate and formic acid were acquired from Sigma Aldrich (St. Louis, MO, USA). Sinopec Jiujiang Branch (Jiujiang, China) provided the petroleum asphalt (Grade 70).

### 2.2. Preparation of Catalysts

#### 2.2.1. Preparation of Porous Carbon (PC)

**Scheme 1:**
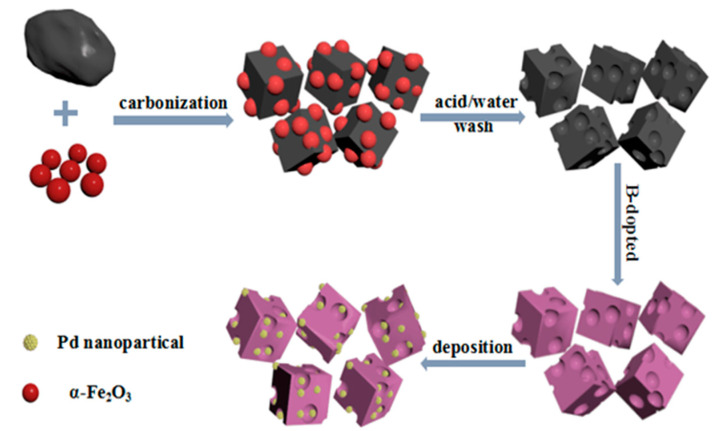
Schematic illustration of the preparation procedure of Pd/BPC catalysts.

PC was prepared through a template-assisted method. As shown in Scheme 1, 2 g of petroleum asphalt was added in 30 mL of toluene, followed by ultrasonic treatment for 10 min. Subsequently, 8 g of α-Fe_2_O_3_ nanoparticles with an average size of 30 nm were introduced into the mixture with continuous stirring. Toluene was then distilled and recovered through vacuum distillation at 50 °C. The collected powder was loaded onto a quartz boat and placed into a tube furnace under a nitrogen atmosphere. Subsequently, carbonization was conducted at 800 °C for 1 h with a heating speed of 5 °C/min. To remove the Fe_2_O_3_ template, the sample was thoroughly washed with hydrochloric acid and DI water several times until no Fe^2+^ or Fe^3+^ ions were detected in the solution. The petroleum asphalt-derived porous carbon was obtained after being dried overnight at 80 °C under vacuum conditions. 

#### 2.2.2. Preparation of Boron-Doped Porous Carbon (BPC)

The obtained PC and boron acid were mixed with a ratio of 1:5 (or 1:3 and 1:7) and calcined at 900 °C (or 800 °C and 1000 °C) in the tube furnace under a nitrogen atmosphere for 1 h with a heating speed of 5 °C/min. After cooling down to room temperature, the BPC support was obtained.

#### 2.2.3. Preparation of Boron-Doped Porous Carbon-Supported Palladium Catalyst (Pd/BPC)

The palladium acetate precursor and the prepared boron-doped carbon material were mixed with a ratio of 1:9 in a methanol solution (15 mL), and the reduction reaction was vigorously stirred in an oil bath at 60 °C (or 0 °C, 20 °C, 40 °C, and 80 °C) for 5 h. After the reaction, filtration and washing with multi-DI water were performed to remove the remaining Pd^2+^ that had not been reduced on the surface and excess methanol. The Pd/BPC catalyst was finally obtained after being dried overnight at 80 °C under vacuum conditions. For comparison, nitrogen-doped porous carbon-supported palladium (Pd/NPC) catalysts was prepared by using a similar procedure except for changing boron acid to urea.

### 2.3. Characterization

The morphology, elements, and particle size distribution of the samples were investigated by scanning electron microscopy (SEM, Hitachi S-4800, Tokyo, Japan), transmission electron microscopy (HR-TEM, FEI-Talos F200X, Hillsboro, OR, USA), and energy-dispersive X-ray spectroscopy (EDX). The X-ray diffraction (XRD) was conducted on a Rigaku Ultima IV X-ray diffractometer with a Cu K source (40 kV, 40 mA). The X-ray photoelectron spectroscopy (XPS) analyses were recorded on an Escalab 250Xi instrument (Chestnut Ridge, Latrobe, PA, USA). The N_2_ adsorption–desorption isotherms were obtained at 77 K using automatic volumetric adsorption equipment (Belsorp-max, Osaka, Japan). After purging the reactor with nitrogen three times, the dehydrogenation of formic acid was carried out, and the generated gas was collected before being analyzed using a gas chromatograph (GC).

### 2.4. Formic Acid’s Dehydrogenation Performance

Briefly, 30 mg of Pd/BPC catalyst and 10 mL of deionized water were placed in a glass bottle reactor, which was placed in a 50 °C stable water bath. The gas was collected and measured using the drainage method to reflect the catalytic performance. The 250 mL water-filled measuring cylinder was inverted in the tank, with its open end positioned below the liquid level of the tank. Additionally, the outlet of the exhaust pipe was fixed at a level equivalent to that of the liquid inside the tank, ensuring equilibrium between internal reactor pressure and atmospheric pressure. When the liquid level in the measuring cylinder was stable and unchanged, 1 mL of sodium formate solution with a concentration of 5 mol/L was measured with a pipette gun and placed in the reactor to ensure the airtight of the system. The gas volume was recorded every 5 min from the time the first bubble appeared. After the reaction, the catalyst was recovered through filtering and used in subsequent cycles.

## 3. Results and Discussions

**Figure 1:**
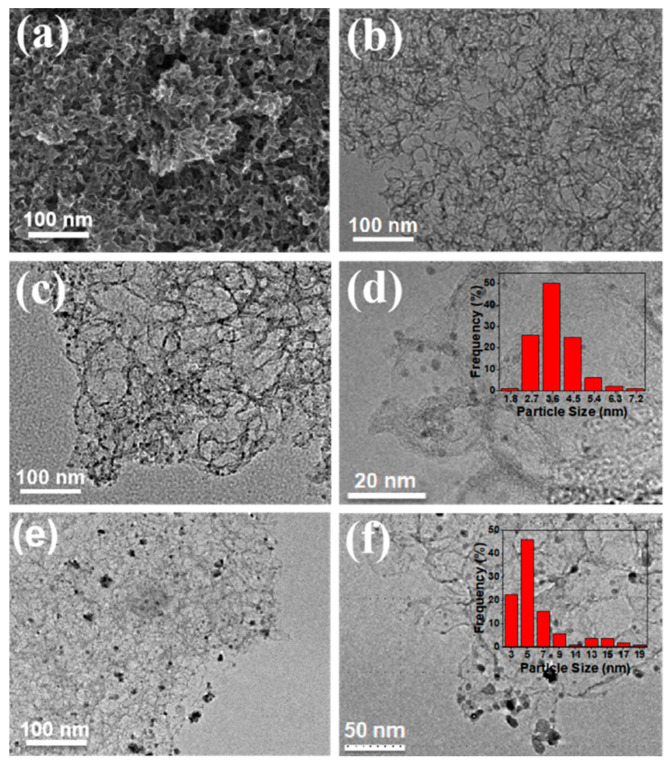
(**a**) SEM and (**b**) TEM images of BPC. (**c**) TEM images of Pd/BPC and (**d**) the corresponding palladium particle-size distribution (inset). (**e**) TEM images of Pd/PC and (**f**) the corresponding palladium particle-size distribution (inset).

The morphologies of microstructures of petroleum asphalt, PC, and BPC were monitored by SEM and TEM images. The petroleum asphalt precursor exhibits a large block morphology with a smooth and uniform surface, displaying no discernible pore structure, as depicted in Figure S1a. In contrast, the SEM image presented in Figure S1b illustrates the porous nature of the carbon material after template carbonization, clearly indicating the successful creation of holes through Fe_2_O_3_ templating [18]. Furthermore, boron doping has a negligible impact on pore formation, as demonstrated in Figure 1a,b. The prepared Pd/BPC catalyst was further examined using TEM analysis. As depicted in Figure 1c, the carbon support shows an abundant pore structure, indicating the effective role played by the template agent, which is consistent with the SEM results. The surface of the prepared Pd/BPC catalyst exhibits fine Pd nanoparticles, with an average size of 3.6 nm, as shown in Figure 1d and Figure S2a, confirming the successful loading of Pd nanoparticles on the porous carbon support. Compared with Pd/PC (Figure 1e,f and Figure S2b), a significant reduction in the size of Pd particles and homogenous distribution can be observed in Pd/BPC, indicating that the incorporation of the B element efficiently facilitates the uniform deposition of the Pd nanoparticles [19]. Additionally, the elemental mappings in Figure S3 demonstrate the homogeneous distribution of Pd, B, and C elements throughout the catalyst’s skeleton.

**Figure 2:**
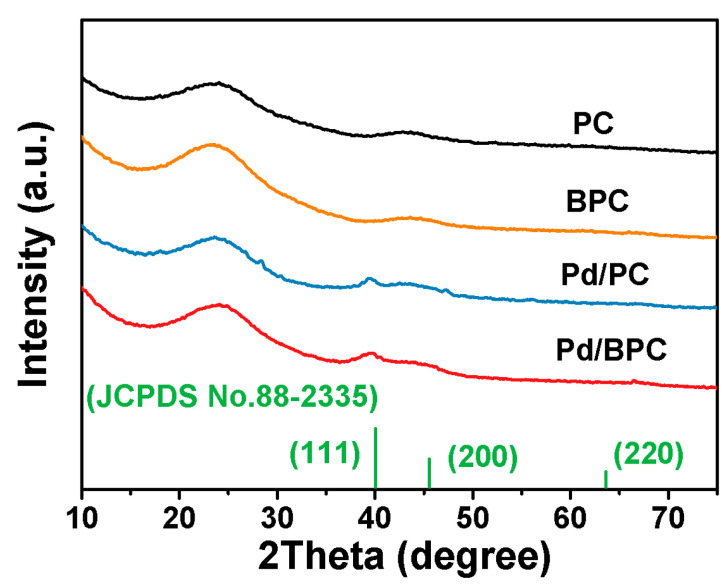
XRD patterns of PC, BPC, Pd/PC, and Pd/BPC.

The catalyst’s structure was further characterized using XRD analysis (Figure 2). The XRD pattern of the porous carbon is depicted by the black curve, indicating an amorphous carbon structure lacking well-defined crystal morphology. Upon introduction of the B element, as evidenced by the orange curve in the XRD pattern, it can be observed that the peaks and valleys become more distinct and diminished, implying a structural modification in the boron-doped porous carbon material. This change can be attributed to the successful incorporation of B atoms, which disrupts the original ordered arrangement and induces greater disorder. The blue and red curves represent the spectra of Pd/PC and Pd/BPC catalysts, respectively. Referring to the standard card of Pd (JCPDS No.88-2335), it can be observed that the positions at 40.1°, 46.7°, and 68.1° in the curves correspond to the characteristic peaks of Pd (111), (200), and (220) crystal planes, respectively [20]. These results demonstrate the successful synthesis of Pd nanoparticles onto the BPC support.

**Figure 3:**
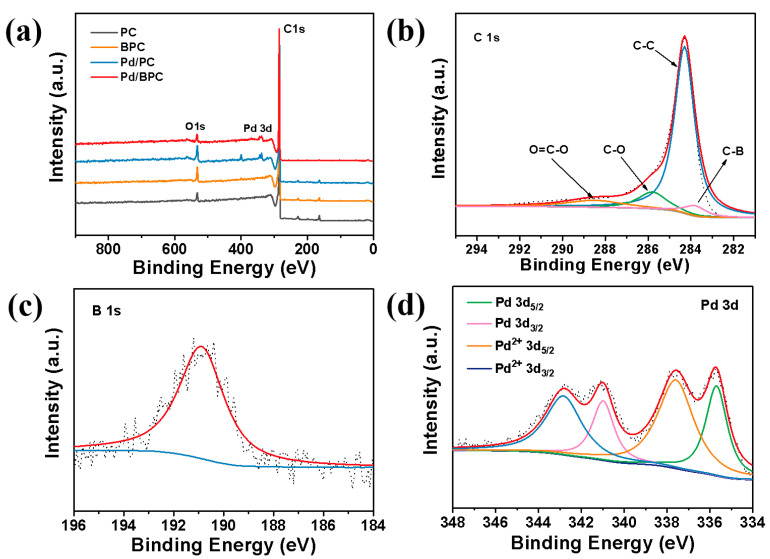
(**a**) Full-range XPS survey spectra of PC, BPC, Pd/PC, and Pd/BPC. (**b**) C 1s, (**c**) B 1s, (**d**) Pd 3d high-resolution XPS spectra of Pd/BPC.

Based on XPS analysis results of Pd/BPC, the material consists of four elements: C, O, B, and Pd (Figure 3). The presence of C and O should originate from the petroleum asphalt precursor. The successful doping of boron is further confirmed by the presence of a B element signal peak, with a mass fraction of 1.3% according to quantitative analysis. The high-resolution C 1s XPS spectrum can be deconvolved into four distinct peaks attributed to C-B, C-C, C-O, and O=C-O functionalities, with peak positions centered at 283.8, 284.2, 285.9, and 288.6 eV, respectively [21]. The appearance of C-B bonds provides evidence that boron atoms are incorporated into the carbon skeleton. In the XPS spectrum of Pd 3d, two pairs of characteristic peaks are observed; specifically, peaks at 335.7 eV and 341.0 eV correspond to Pd(0), while peaks at 337.2 eV and 342.5 eV correspond to Pd^2+^ [22]. The observation indicates the coexistence of two forms of Pd: zero-valent and bivalent, implying an electronic interaction with the support. Quantitative analysis shows that the mass fraction of Pd in Pd/BPC is about 3.0 wt.%.

**Figure 4:**
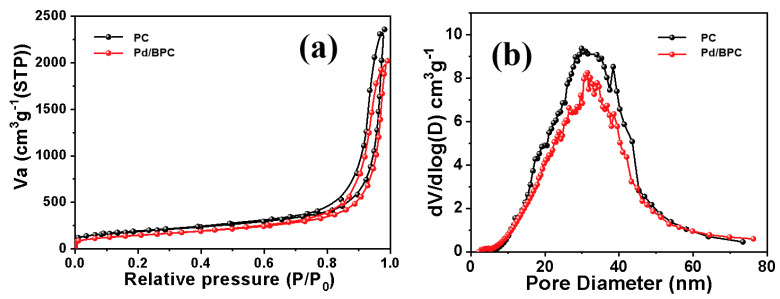
(**a**) N_2_ adsorption–desorption isotherms, (**b**) pore size distribution curves of PC and Pd/BPC.

The pore structures of the carbon support and Pd/BPC catalyst were investigated using N_2_ adsorption–desorption measurements. According to the N_2_ adsorption–desorption isotherms (Figure 4a), the BET-specific surface area (S_BET_) of PC was determined to be 663.5 m^2^ g^−1^. Upon loading with Pd nanoparticles and doping with boron, the S_BET_ slightly decreases to 530.1 m^2^ g^−1^ in the Pd/BPC catalyst. Analysis of the pore size distribution revealed that PC possesses a mesoporous structure with a diameter centered at 27.3 nm and pore volume of 3.52 cm^3^ g^−1^, while Pd/PBC exhibits a slightly decreased mesoporous structure with a diameter centered at 26.3 nm and pore volume of 3.03 cm^3^ g^−1^ (Figure 4b), indicating successful immobilization of Pd nanoparticles within the carbon pores [23].

To investigate the impact of the boron doping amount on the catalyst performance, we synthesized three samples of boron-doped porous carbon with varying ratios of carbon-to-boron source (1:3, 1:5, and 1:7) during the doping step. Additionally, a control sample consisting of non-boron-doped carbon material was prepared. Subsequently, their catalytic performance in the dehydrogenation of formic acid was evaluated, as presented in Figure S4. It can be seen that the incorporation of boron significantly enhances the catalytic performance compared with the undoped Pd/PC catalyst. Within a certain range, an increase in boron content leads to improved performance for the Pd/BPC catalysts. It is noteworthy that there is minimal difference between the 1:5 and 1:7 ratio, indicating a saturation point beyond which further increase in boron content has a negligible impact on the catalytic performance. Considering the faster hydrogen production rate and cost-effectiveness observed at an early stage, a carbon-to-boron ratio of 1:5 was determined to be the optimal choice.

Subsequently, to investigate the impact of doping temperature on catalyst performance, three sets of comparative experiments were conducted by calcining a mixture of boric acid and porous carbon at temperatures of 800 °C, 900 °C, and 1000 °C, respectively. The experimental results demonstrate that the Pd/BPC_900_ catalyst prepared at 900 °C exhibited superior performance, followed by 1000 °C, while significantly lagging at 800 °C (Figure S5). It is hypothesized that the effective incorporation of B atoms into the stable structure of porous carbon is hindered at low temperatures. As the temperature increases, the integrity of the six-membered ring structure gradually weakens, allowing for the successful incorporation of B atoms. Nevertheless, further temperature elevation would result in the decomposition of both boric acid and boron-doped porous carbon, rendering them unstable entities.

Furthermore, Pd nanoparticles are loaded onto BPC support by employing anhydrous methanol as a mild reducing agent, and the reduction temperature significantly influences the chemical valence state and proportion of the Pd nanoparticles. A series of comparative experiments were conducted at 0 °C, 20 °C, 40 °C, 60 °C, and 80 °C, respectively. Correspondingly, five Pd/BPC catalysts were prepared and their catalytic performance was evaluated. The results presented in Table S1 demonstrate that the higher reduction temperature generally enhances the overall catalyst performance, whereas a decline in effectiveness is observed beyond 60 °C. The weak reducing nature of methanol leads to a pronounced reduction effect on Pd^2+^ at elevated temperatures, with the degree of reduction increasing proportionally with temperature. However, the reaction system becomes highly unstable when the reduction temperature exceeds the boiling point of methanol (64.7 °C) [24], leading to ineffective support for reduced Pd nanoparticles on the porous carbon support. Therefore, the optimal reduction temperature for catalyst preparation was determined to be 60 °C.

**Figure 5:**
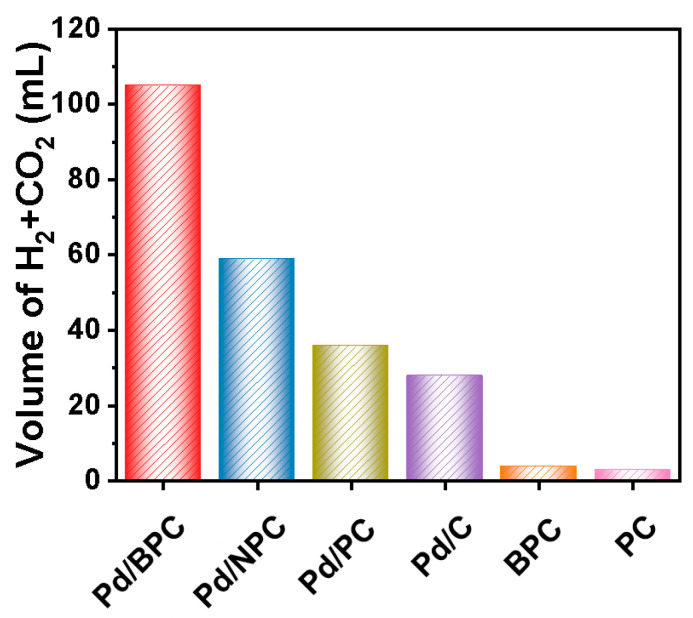
Comparison of the catalytic performance in formic acid dehydrogenation over Pd/BPC, Pd/NPC, Pd/PC, Pd/C, BPC, and PC.

To investigate the significant role of boron doping on the performance of Pd, a comprehensive comparative analysis was conducted using various samples including commercially available palladium carbon (Pd/C), Pd/PC, and Pd/NPC catalysts, as well as the BPC and PC supports. This study demonstrates the superior catalytic performance achieved by the Pd/BPC catalyst, as shown in Figure 5 below. The BPC support showed slightly higher activity than that of pure PC, although both of them showed poor activity for the dehydrogenation of formic acid without the presence of Pd. At a low reaction temperature of 50 °C, the commercial Pd/C could facilitate the decomposition of formic acid and deliver about 27 mL of mixed H_2_ and CO_2_ in 90 min. By loading Pd nanoparticles on the petroleum asphalt-derived porous carbon, an increased volume of gas (36 mL) could be obtained over the Pd/PC catalyst. Remarkably, the Pd/BPC catalyst was shown to be capable of yielding an impressive 105 mL of H_2_ and CO_2_ without producing poisonous CO gas; this yield is about 2.9 and 3.8 times higher than that of the Pd/PC and commercial Pd/C, respectively. As a control, the Pd/NPC catalyst was also prepared with the same method by introducing nitrogen to the support, which produced 58 mL of mixed gas in the same space of time, thus surpassing the catalytic performance of Pd/PC but remaining far behind that of Pd/BPC. Comparatively, the results revealed that the Pd/BPC catalyst displayed a superior hydrogen production rate, greatly surpassing other tested catalysts. This outstanding achievement highlights the significant promotional effect demonstrated by boron-doped Pd catalysts in enhancing catalytic activity [25].

**Figure 6:**
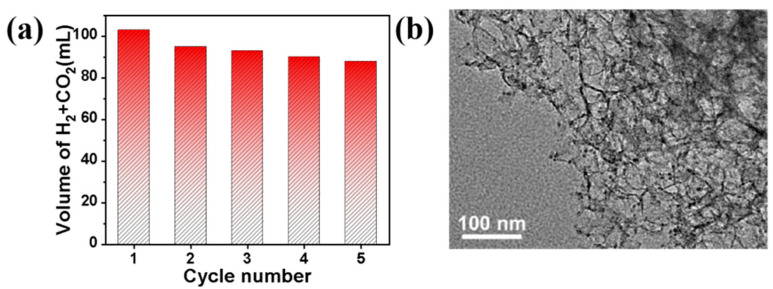
(**a**) The recyclability of Pd/BPC for formic acid dehydrogenation; (**b**) TEM image of Pd/BPC after being used for five cycles.

The cycle stability of the catalyst is a crucial characteristic, and we conducted cycling tests to evaluate the stability of the Pd/BPC catalyst. Every recovery operation causes some loss of the catalyst in the filtration process, leading to a slight reduction in corresponding catalytic performance. However, it is noteworthy that this trend remains within an acceptable range (Figure 6a). As can be seen, the catalyst showed excellent recyclability with no significant reduction after five consecutive cycles. The TEM image additionally revealed that there was no significant accumulation of Pd nanoparticles on the catalyst surface, indicating excellent catalytic stability (Figure 6b). The reinforced catalytic performance and durability of Pd/BPC can be attributed mainly to the introduction of boron into the palladium–porous carbon catalyst. Boron doping can tailor the electronic properties of palladium nanoparticles by modifying the surface structure and electronic density distribution of the support. This modification would lead to improved adsorption and activation of reactant molecules on the catalytic surface [26]. Additionally, the incorporation of B atoms leads to a homogenous distribution of Pd nanoparticles and would prevent the agglomeration and sintering of palladium nanoparticles during catalysis, ensuring long-term stability and sustained high activity levels are maintained [27]. Moreover, compared to other commonly used carbon supports such as activated carbon, porous carbon (PC), and nitrogen-doped porous carbon (NPC), BPC offers unique advantages for supporting palladium nanoparticles due to its highly ordered mesoporous structure, which provides a large specific surface area and abundant accessible active sites for efficient reactant diffusion and interaction with the metal nanoparticles [28].

**Figure 7:**
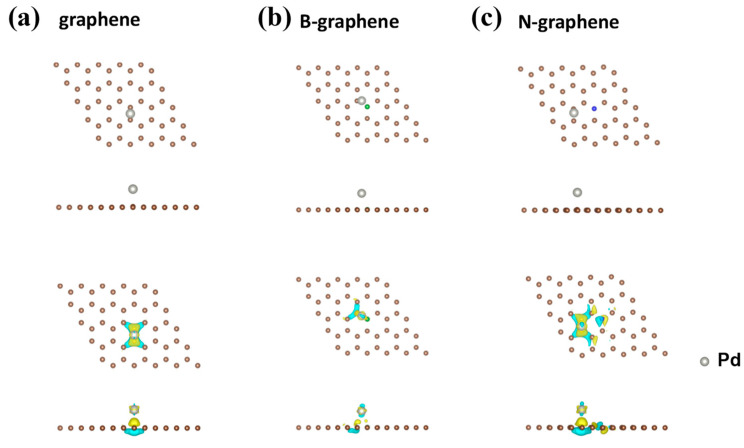
Structural models and difference charge density of Pd loading onto (**a**) graphene, (**b**) N-graphene, and (**c**) B-graphene substrates. Yellow and blue represent electron gain and loss, respectively.

To elucidate the positive impact of boron doping on the catalytic performance of Pd in more depth, density functional theory (DFT) calculations were further conducted. We built graphene, B-graphene, and N-graphene models as substrates and calculated the adsorption energy (E_ad_) of Pd atoms onto them. According to the DFT results shown in Figure 7, the adsorption energy of Pd on B-graphene (−1.10 eV) is significantly higher than that of N-graphene (−0.49 eV) and undoped graphene (−0.47 eV), which provides a crucial insight into the exceptional properties of the Pd/B-graphene model. Graphene itself possesses advantages such as excellent electrical conductivity and high specific surface area, providing sufficient vacancies to accommodate metal catalyst particles; however, the absence of additional elements restricts the bonding strength and adsorption energy between the metal catalyst and substrate [29]. As for the N-graphene model, the introduction of N atoms induces an augmentation of charge density on surrounding C atoms, which can even promote electronic interaction between the Pd and C atoms on the substrate, resulting in the enhanced catalytic performance of Pd/NPC [30,31]. Remarkably, after incorporating B atoms into the crystal lattice of carbon, the B-C structure exhibits a greater number of surface active sites and stronger chemical reactivity than pure C [32]. More importantly, as supported by the observed difference in charge density, the strong charge transfer effect between B and C atoms further enables effective regulation of the metal–support interaction. Consequently, in the Pd/B-graphene system, a tighter, more stable, and durable bond forms between Pd and the boron-doped carbon substrate. Such an interaction leads to an appropriate reduction of the electron density of Pd 3d orbitals, significantly strengthening the catalytic activity for formic acid dehydrogenation [21]. It can also be seen from the partial density of states (DOS) in Figure S6 that the band gap of carbon atoms near the Fermi surface is widened due to the introduction of boron and nitrogen atoms. Combining the experimental and theoretical results, boron doping not only facilitates the homogenous distribution of Pd nanoparticles but also induces a dramatic support–metal interaction, accounting for the reinforced performance of Pd/BPC.

## 4. Conclusions

In summary, a palladium catalyst supported on a boron-doped porous carbon catalyst (Pd/BPC) was successfully prepared through a template-assisted method by employing anhydrous methanol as a mild reducing agent. The introduction of boron can modulate the property of the carbon substrate while preserving its porous structure and high specific surface area, which not only facilitates the homogenous distribution of Pd nanoparticles but also induces a stronger support–metal interaction to optimize the electronic density of Pd catalyst, thereby reinforcing the catalytic performance. The optimal Pd/BPC catalyst synthesized with a carbon-to-boron ratio of 1:5 by calcination at 900 °C and subsequent reduction at 60 °C exhibited superior catalytic performance in the dehydrogenation of formic acid, being 2.9 and 3.8 times greater than that of the Pd/PC and commercial Pd/C, respectively. The catalyst also possessed good stability, showing no significant reduction in activity after five consecutive cycles. This work paves the way for the synthesis of highly active supported Pd catalysts to be used in formic acid decomposition for hydrogen production.

## Data Availability

Data are contained within the article and Supplementary Materials.

## Supplementary Materials

The following supporting information can be downloaded at: https://www.mdpi.com/article/10.3390/nano14060549/s1, Figure S1: SEM images of petroleum asphalt (a) and PC (b); Figure S2: HRTEM images of (a) Pd/BPC and (b) Pd/PC; Figure S3: TEM image of Pd/BPC and corresponding EDS mapping for the C, B, and Pd elements; Figure S4: The catalytic performance of Pd/BPC_r_ (r = 3, 5, 7) for dehydrogenation of formic acid; Figure S5: The catalytic performance of Pd/BPC_T_ (T = 800, 900, 1000 °C) for dehydrogenation of formic acid; Table S1: The catalytic performance of Pd/BPC_t_ (t = 0, 20, 40, 60, 80 °C) for the dehydrogenation of formic acid; Figure S6: The partial density of states (DOS) of (a) graphene, (b) N-graphene, and (c) B-graphene.

## References

[1] Jiang K., Xu K., Zou S., Cai W.-B. (2014). B-Doped Pd catalyst: Boosting room-temperature hydrogen production from formic acid–formate solutions. J. Am. Chem. Soc..

[2] Hossain M.R., Singh S., Sharma G.D., Apostu S.-A., Bansal P. (2023). Overcoming the shock of energy depletion for energy policy? Tracing the missing link between energy depletion, renewable energy development and decarbonization in the USA. Energy Policy.

[3] Li J., Chen W., Zhao H., Zheng X., Wu L., Pan H., Zhu J., Chen Y., Lu J. (2017). Size-dependent catalytic activity over carbon-supported palladium nanoparticles in dehydrogenation of formic acid. J. Catal..

[4] Osman A.I., Mehta N., Elgarahy A.M., Hefny M., Al-Hinai A., Al-Muhtaseb A.A.H., Rooney D.W. (2022). Hydrogen production; storage, utilisation and environmental impacts: A review. Environ. Chem. Lett..

[5] Enthaler S., von Langermann J., Schmidt T. (2010). Carbon dioxide and formic acid—The couple for environmental-friendly hydrogen storage?. Energy Environ. Sci..

[6] Cao T., Cheng J., Ma J., Yang C., Yao M., Liu F., Deng M., Wang X., Ren Y. (2021). Facile Synthesis of Microporous Carbons from Biomass Waste as High Performance Supports for Dehydrogenation of Formic Acid. Nanomaterials.

[7] Sun X.F., Ding Y.Y., Feng G., Yao Q.L., Zhu J., Xia J.H., Lu Z.H. (2023). Carbon bowl-confined subnanometric palladium-gold clusters for formic acid dehydrogenation and hexavalent chromium reduction. J. Colloid Interface Sci..

[8] Fellay C., Dyson P.J., Laurenczy G. (2008). A Viable Hydrogen-Storage System Based On Selective Formic Acid Decomposition with a Ruthenium Catalyst. Angew. Chem. Int. Ed..

[9] Yaacoub L., Dutta I., Werghi B., Chen B.W.J., Zhang J., Hamad E.A., Ang E.P.L., Pump E., Sedjerari A.B., Huang K.W. (2022). Formic Acid Dehydrogenation via an Active Ruthenium Pincer Catalyst Immobilized on Tetra-Coordinated Aluminum Hydride Species Supported on Fibrous Silica Nanospheres. ACS Catal..

[10] Rahman M.M., Dutta I., Chakraborty P., Alobaid N.A., Gholap S.S., Rachuri Y., Alrais L., Huang K.-W. (2023). Selective hydrogen generation from formic acid catalyzed by an iridium-azocarboxamide complex under neat conditions. ARKIVOC.

[11] Alrais L., Gholap S.S., Dutta I., Abou-Hamad E., Chen B.W.J., Zhang J., Hedhili M.N., Basset J.-M., Huang K.-W. (2024). Highly efficient immobilized PN3P-pincer iridium catalyst for dehydrogenation of neat formic acid. Appl. Catal. B Environ..

[12] Ruiz-López E., Peláez M.R., Ruz M.B., Leal M.I.D., Tejada M.M., Ivanova S., Centeno M.A. (2023). Formic Acid Dehydrogenation over Ru- and Pd-Based Catalysts: Gas- vs. Liquid-Phase Reactions. Materials.

[13] Poudel M.B., Vijayapradeep S., Sekar K., Kim J.S., Dong J.Y. (2024). Pyridinic-N exclusively enriched CNT encapsulated NiFe interfacial alloy nanoparticles on knitted carbon fiber cloth for bifunctional oxygen catalysts and biaxially flexible zinc-air batteries. J. Mater. Chem. A.

[14] Koh K., Jeon M., Yoon C.W., Asefa T. (2017). Formic acid dehydrogenation over Pd NPs supported on amine-functionalized SBA-15 catalysts: Structure–activity relationships. J. Mater. Chem. A.

[15] Li Z., Yang X., Tsumori N., Liu Z., Himeda Y., Autrey T., Xu Q. (2017). Tandem Nitrogen Functionalization of Porous Carbon: Toward Immobilizing Highly Active Palladium Nanoclusters for Dehydrogenation of Formic Acid. ACS Catal..

[16] Kim Y., Kim D.H. (2020). Hydrogen production from formic acid dehydrogenation over a Pd supported on N-doped mesoporous carbon catalyst: A role of nitrogen dopant. Appl. Catal. A Gen..

[17] Lin Z., Liu O., Guan S., Zhao X., Yuan Z., Liu X., Bian L., Fan Y., Peng Q., Han S. (2023). Self-crosslinked N-doped carbon dot supported Pd as an efficient catalyst for dehydrogenation of formic acid at room temperature. Sustain. Energy Fuels.

[18] Shi J., Cui H., Xu J., Yan N., Liu Y. (2020). Design and fabrication of hierarchically porous carbon frameworks with Fe_2_O_3_ cubes as hard template for CO_2_ adsorption. Chem. Eng. J..

[19] Wang J.-Y., Kang Y.-Y., Yang H., Cai W.-B. (2009). Boron-doped palladium nanoparticles on carbon black as a superior catalyst for formic acid electro-oxidation. J. Phys. Chem. C.

[20] Liu S., Zhang H., Ren T., Yu H., Deng K., Wang Z., Xu Y., Wang L., Wang H. (2023). Interface Engineering and Boron Modification of Pd-B/Pd Hetero-Metallene Synergistically Accelerate Oxygen Reduction Catalysis. Small.

[21] Xia Y., Zhao X., Xia C., Wu Z.-Y., Zhu P., Kim J.Y., Bai X., Gao G., Hu Y., Zhong J. (2021). Highly active and selective oxygen reduction to H_2_O_2_ on boron-doped carbon for high production rates. Nat. Commun..

[22] Kim Y., Kim S.H., Ham H.C., Kim D.H. (2020). Mechanistic insights on aqueous formic acid dehydrogenation over Pd/C catalyst for efficient hydrogen production. J. Catal..

[23] Yang P., Zhang L., Wei X., Dong S., Cao W., Ma D., Ouyang Y., Xie Y., Fei J. (2023). “Special” Solvent to Prepare Alloyed Pd_2_Ni_1_ Nanoclusters on a MWCNT Catalyst for Enhanced Electrocatalytic Oxidation of Formic Acid. Nanomaterials.

[24] Badawy T., Xu H., Li Y. (2022). Macroscopic spray characteristics of iso-octane, ethanol, gasoline and methanol from a multi-hole injector under flash boiling conditions. Fuel.

[25] Li H., Qin X., Zhang X.-G., Jiang K., Cai W.-B. (2022). Boron-Doped Platinum-Group Metals in Electrocatalysis: A Perspective. ACS Catal..

[26] Kumar S.S., Himabindu V. (2020). Boron-Doped Carbon nanoparticles supported palladium as an efficient hydrogen evolution electrode in PEM water electrolysis. Renew. Energy.

[27] Jiang T., Yu L.Y., Zhao Z.J., Wu W., Wang Z.C., Cheng N.C. (2022). Regulating the intermediate affinity on Pd nanoparticles through the control of inserted-B atoms for alkaline hydrogen evolution. Chem. Eng. J..

[28] Su Y., Yao C., Zhang Q., Xu L., Wang H., Liu J., Hou S. (2019). Palladium Nanoparticles Supported on B-Doped Carbon Nanocage as Electrocatalyst toward Ethanol Oxidation Reaction. ChemElectroChem.

[29] Feng J.R., Wang G.C. (2021). Theoretical insight into the role of nitrogen in the formic acid decomposition over Pt13/N-GNS. Appl. Surf. Sci..

[30] Bi Q.Y., Lin J.D., Liu Y.M., He H.Y., Huang F.Q., Cao Y. (2016). Dehydrogenation of Formic Acid at Room Temperature: Boosting Palladium Nanoparticle Efficiency by Coupling with Pyridinic-Nitrogen-Doped Carbon. Angew. Chem..

[31] Huang Y.Y., Pan P.B., Li Q.H., Han B.Y., Ye R.P., Yao Y.G. (2023). Regulating the metal-support interactions by tuning the ratios between N and B based on the C2N motif to develop efficient Pd-based catalysts for CO oxidative coupling to DMO: A DFT study. Appl. Surf. Sci..

[32] Ibarra-Prieto H.D., Garcia-Garcia A., Aguilera-Granja F., Navarro-Ibarra D.C., Rivero-Espejel I. (2023). One-Pot, Optimized Microwave-Assisted Synthesis of Difunctionalized and B-N Co-Doped Carbon Dots: Structural Characterization. Nanomaterials.

