# Supplementary material for: A Palladium Catalyst Supported on Boron-Doped Porous Carbon for Efficient Dehydrogenation of Formic Acid
Source: Nanomaterials (Basel). 2024 Mar 20;14(6):549. doi: 10.3390/nano14060549 (PMC10975864; doi:10.3390/nano14060549)
Supplement: Supplementary file 1 [file nanomaterials-14-00549-s001.zip › nanomaterials-2908145-supplementary.pdf]

Palladium Catalyst Supported on Boron-Doped Porous Carbon for Efficient Dehydrogenation of Formic Acid

Hui Liu[1], Mengyuan Huang[1], Wenling Tao[1], Liangliang Han[1], Jinqiang Zhang[3] and Qingshan Zhao[2,*]

[1] College of Chemistry and Chemical Engineering, Yantai University, Yantai 264005, China
[2] State Key Laboratory of Heavy Oil Processing, College of Chemistry and Chemical Engineering, China University of Petroleum (East China), Qingdao 266580, China
[3] School of Chemical Engineering, The University of Adelaide, North Terrace, Adelaide, SA 5005, Australia
* Correspondence: qszhao@upc.edu.cn

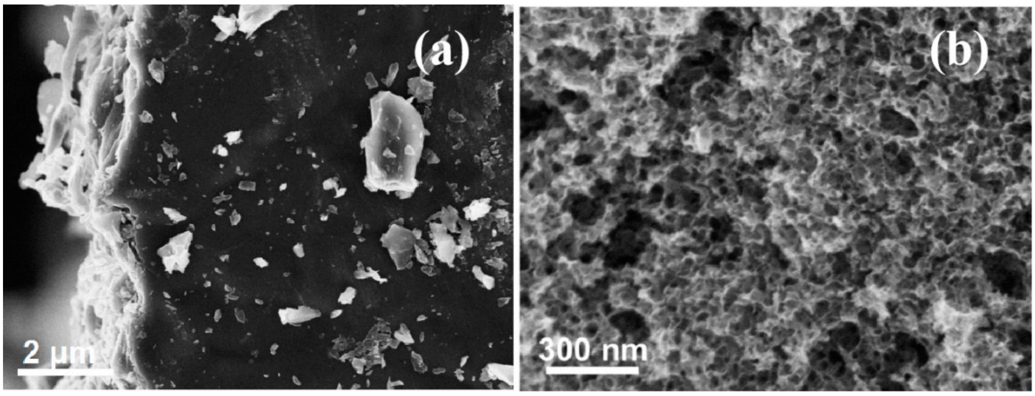

Figure S1. SEM images of petroleum asphalt (a) and PC (b).

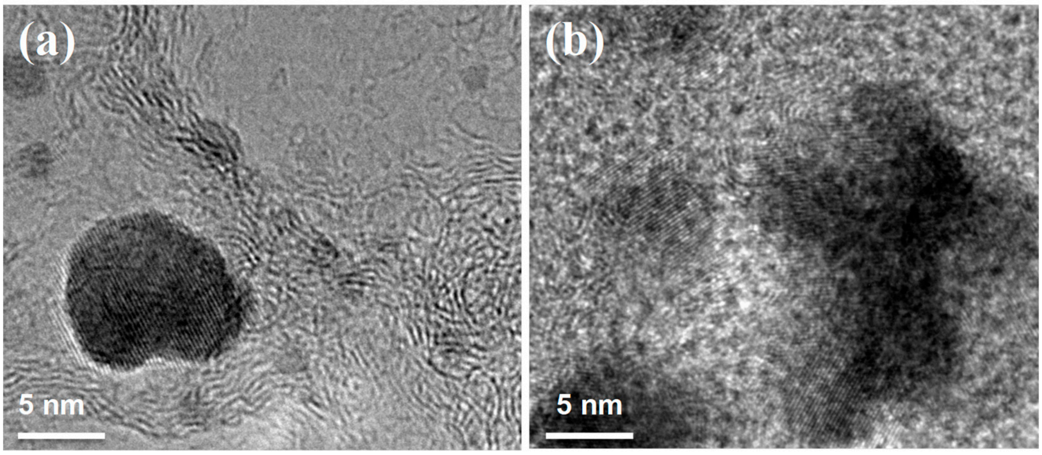

Figure S2. HRTEM images of (a) Pd/BPC and (b) Pd/PC.

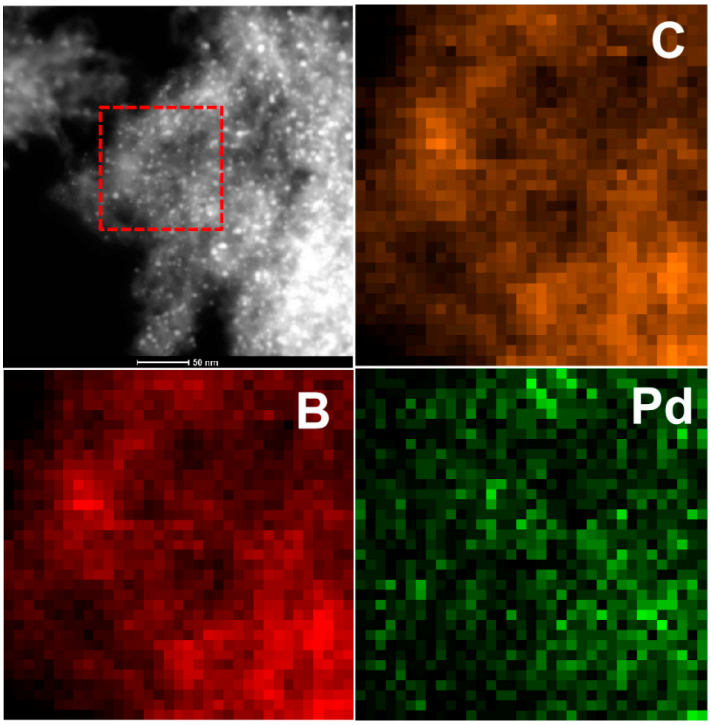

Figure S3. TEM image of Pd/BPC and corresponding EDS mapping for the C, B, and Pd elements.

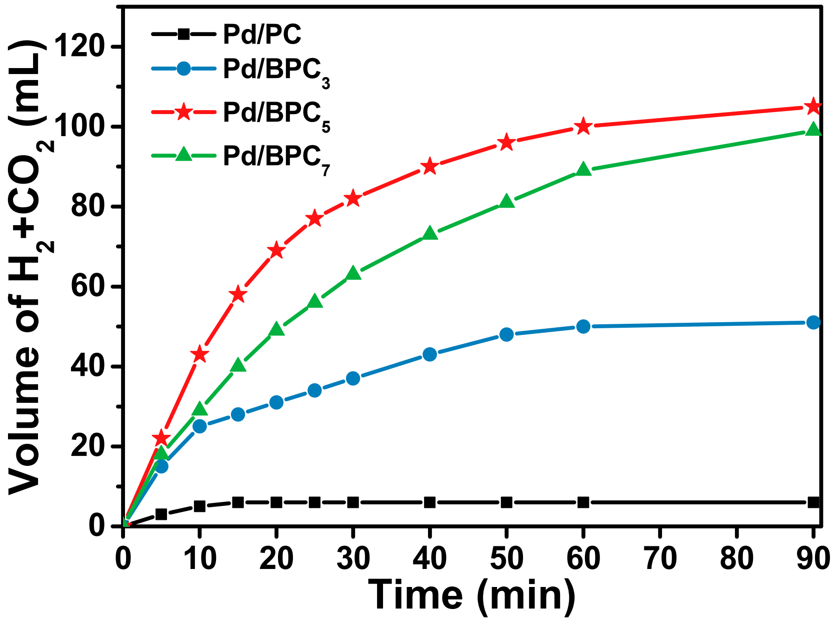

Figure S4. The catalytic performance of Pd/BPC$_r$ (r=3, 5, 7) catalysts for dehydrogenation of formic acid.

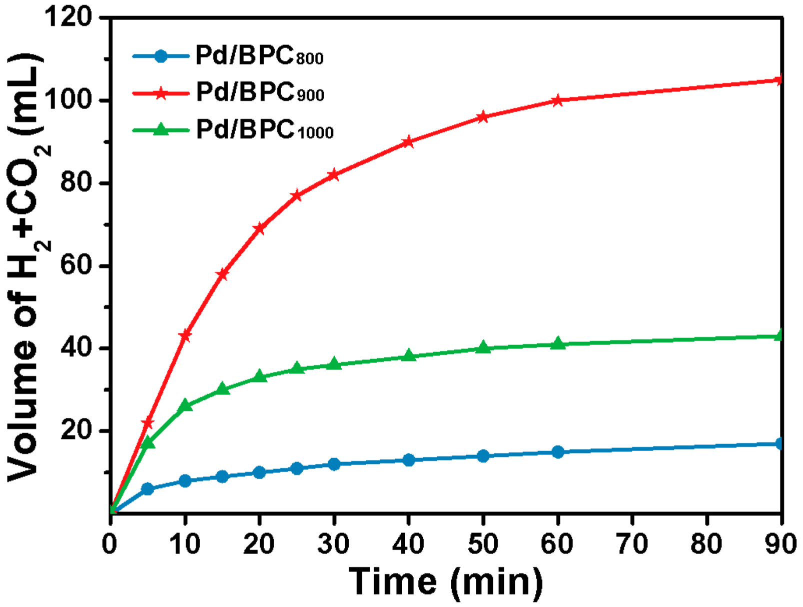

Figure S5. The catalytic performance of Pd/BPCT (T=800, 900, 1000 °C) for dehydrogenation of formic acid.

Table S1. The catalytic performance of Pd/BPC_t (t=0, 20, 40, 60, 80 ºC) for dehydrogenation of formic acid.

**Entry**	**Catalyst**	**Reduction temperature (ºC)**	**V_{gas} (mL)**
1	Pd/BPC	0	8
2	Pd/BPC	20	18
3	Pd/BPC	40	36
4	Pd/BPC	60	105
5	Pd/BPC	80	73

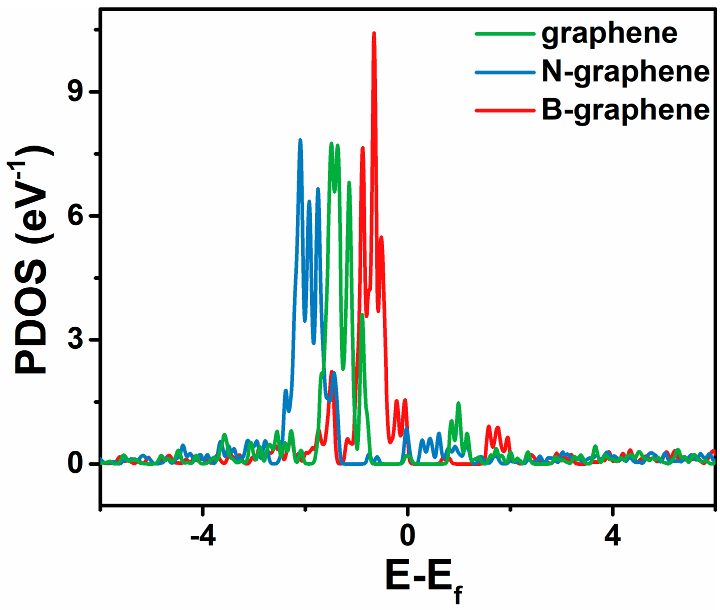

Figure S6. The partial DOS of (a) graphene, (b) N-graphene, and (c) B-graphene.
